# The Multiple Forms of *Leishmania major* in BALB/C Mice Lung in Iran

**Published:** 2012

**Authors:** SH Shirbazou, M Jafari

**Affiliations:** 1 Department of Parasitology, Faculty of Medicine, Baqiyatallah University of Medical Sciences, Tehran, Iran; 2 Research Center of Molecular Biology, Baqiyatallah University of Medical Sciences, Tehran, Iran; 3 Department of Biochemistry, Faculty of Medicine, Baqiyatallah University of Medical Sciences, Tehran, Iran

**Keywords:** Multiple forms, *Leishmania major*, BALB/c mice, Lung

## Abstract

Cutaneous leishmaniasis is one of the most important parasitic diseases, which are endemic in different parts of Iran. *Leishmania major* and *L. tropica* are the primary causative agents of this disease. The aim of the present study was to detect the multiple forms of *L. major* in lung. Ppromastigotes of *L. major* at stationary phase were injected to BALB/c mice. After 60 days, the different forms of *Leishmania* parasites were checked in lung tissue. Promastigote and amastigote forms of *Leishmania* parasites were detected.

## Introduction

Leishmaniasis is one of the most important public health problems worldwide that affects 12 million people in 88 countries and threatens an additional 350 million people worldwide (1). It has several different clinical features including cutaneous, visceral, and mucocutaneous. Cutaneous leishmaniasis (CL) is a common parasitic disease in Iran that is mainly caused by two species of *Leishmania tropica* and *L. major*. Annually, there are 1.5-2 million of new cases of cutaneous leishmaniasis (1–3). Parasite properties (infectivity, virulence) and host factors (the genetic and immunological status) regulate various disease expressions. “The face, neck, and arms are the commonest targets, although the location of the lesion in a covered area such as the shins is usual in Iran” (4–5).

*Leishmania* lives extracellularly as flagellated promastigotes in the gut and salivary glands of the sandfly vector and intracellularly as amastigotes in the vertebrate host macrophages. Viscerotropic species of parasite migrates to liver, spleen, and bone marrow and if left untreated it will always result in the death in the host (1, 6). To our knowledge, there are no the report to the presence of *L. major* in BALB/c mice lung. In the present study, we describe the detection of multiple forms of *L. major* in lung.

## Case report

Female BALB/c mice (20–30 g body wt.) were obtained from Pastor Institute (Tehran, Iran). All animal experiments were carried out with the approval of Institutional Animal Ethical Committee. BALB/c mice were selected, because they are very sensitive to *L. major* infection (7). Promastigotes of *L. major* MRHO/IR/75/ER strain were harvested at stationary phase and used to infect BALB/c mice. The base of the tail was injected intradermally with inoculums of 2×10^6^ promastigotes in 0.1 ml. After 4-6 weeks at the injection site, the wound was detected due to parasite growth. After 60 days, the animal was dissected and a few thin smears were prepared from its lung. All prepared smears were fixed by methanol, stained with 10% Giemsa for 10 min, and examined microscopically for the presence of different forms of *L. major*.

**Fig. 1:**
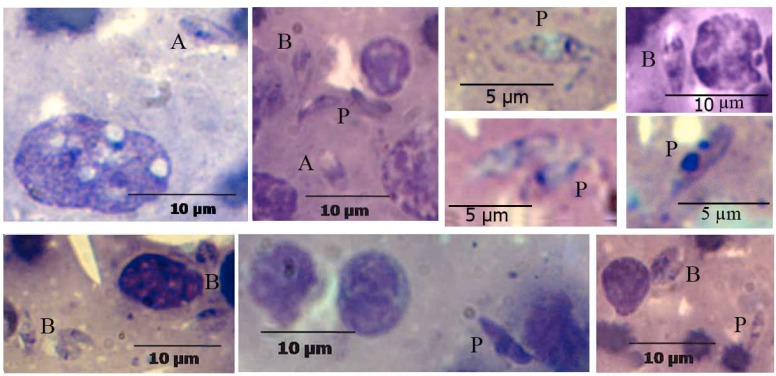
Different forms of *L. major* in the lung of infected BALB/c after 60 days. A: amastigote, P: promastigote and B: binary fission (Gimsa, 1000X)

The different forms of the *Leishmania* parasites were detected in the lung of infected BALB/c. These forms were including the development of the forms divided amastigote into promastigotes and division stages by binary fission (Fig. 1).

## Discussion

CL is an infectious parasitic disease in some areas of Iran. The infective stage of the parasites in the form of promastigotes is injected into the skin of patient by insect bite (8, 9). When the parasite enters the mammalian host inoculated, it undergoes a transformation process, which results in small, round and nonflagellated amastigote form. Amastigotes replicate inside the macrophage, are finally released from the macrophage, and attack other macrophages (10). In the present study, pomastigote and amastigote forms of *Leishmania* parasites were detected in the lung infected BALB/c after 60 days. In a study, *L. tropica* amastigotes were detected in liver and spleen of mucosal and visceral lesions of a puppy (11). In addition, the presence of this parasite has previously been detected in skin of patients with cutaneous visceral leishmaniasis (CVL) and HIV co-infection in Iran (12). However, the majority of cases of HIV–leishmaniasis co-infection reported in the Mediterranean basin were caused by *L. infantum* (13).

The molecular mechanism of *Leishmania* differentiation and the host–parasite molecular interactions in leishmaniasis are not well understood. Evidence suggests that susceptibility to infection was associated with activation of Th2 cells secreting interleukine (IL)-4, IL-5, IL-6 and IL-10 (14). BALB/c mice produce Th2-type cytokines, which is associated with disease progression and susceptibility (6, 7). Cytokines-activated macrophages activate the expression of genes responsible for synthesis of intermediates such as by reactive oxygen and nitrogen intermediates (ROI and RNI), especially NO, which contribute to the regulation of the inflammatory response (6, 15). *Leishmania* pro-mastigotes have been shown to be susceptible to both ROIs and RNIs (15). High concentrations of RNI and ROI have pro-apoptotic effects and prevent the development of parasite (16). NO killed *Leishmania* parasites by inducing amastigotes apoptosis (14). Inside the macrophage, the parasite also resists the killing activity of the macrophage. Biochemical features of the parasite play a pivotal role in parasite survival inside the macrophages (10).

The amastigote-promastigote differentiation speed and growth rate are crucial for the generation of a transmittable parasite population. Acidic environment or elevated temperature changes the promastigote to ama-stigote form (17). Optimal temperature range for propagation *L. major* amastigotes is 33°C to 34°C with pH equal to 3.5 (9). Another study show that NO-resistant amastigotes differentiated more rapidly into promastigotes and have a higher potential of transmission to mammalian hosts than the wild type (14). However, further studies are required for a more comprehensive und-erstanding of biochemical pathways to grow and differentiate of parasite in lung cells.
